# Effect of environmental enrichment for piglets in the nursery phase

**DOI:** 10.5713/ajas.20.0222

**Published:** 2020-06-24

**Authors:** Joselaine Bortolanza Padilha-Boaretto, Priscila Michelin Groff-Urayama, Suelen Maria Einsfeld, Cleverson de Souza, Angélica Signor Mendes, Emilyn Midori Maeda, Sabrina Endo Takahashi

**Affiliations:** 1 Department of Animal Science, Federal University of Technology – Paraná (UTFPR), Campus Dois Vizinhos, Paraná 85660-000, Brazil; 2 School of Veterinary Medicine and Animal Science, São Paulo State University (UNESP), Botucatu, 18610-307, Brazil

**Keywords:** Animal Behaviour, Nursery Pig Breed, Environmental Enrichment, Welfare

## Abstract

**Objective:**

The effect of environmental enrichment on the behaviour of piglets in the nursery phase was evaluated.

**Methods:**

A total of 450 hybrid pigs (21 day old), including both females and uncastrated males, weighing approximately 6 kg, were distributed in a completely randomised design with 3 treatments and 3 replicates of 50 animals each. The treatments were: i) pen without environmental enrichment (control), ii) treatment consisting of continuous environmental enrichment (CEE) with rubber balls throughout the experimental period, and iii) treatment consisting of environmental enrichment with washed balls (EEWB) during the whole experimental period which were removed daily for washing. For the behavioural evaluation, 10 animals were randomly selected per replicate. The behavioural assessments were performed once a week, from 8 am to 6 pm, using images captured with a video camera. The data were submitted to non-parametric analyses, the means were compared using the Bonferroni test, and Person’s correlations were also calculated.

**Results:**

A statistical difference (p = 0.001) was observed in the B5 (playful) behaviour; the animals in the EEWB treatment group had a higher frequency of this behaviour than animals in the control treatment group. The animals in the control group showed a higher frequency of B7 behaviour (lying down) (p = 0.026) than those in the EEWB and CEE treatment groups. The animals in the control group had a higher frequency of the B9 (belly nosing) behaviour than those in the EEWB group (p = 0.015). There was a tendency towards a higher frequency of behaviour B3 (walking in the pen) (p = 0.067) when the animals received the control treatment than treatments EEWB or CEE.

**Conclusion:**

The use of an enriching object improved the animal welfare and altered the correlation between the evaluated behaviours compared to the animals that did not have environmental enrichment.

## INTRODUCTION

Swine breeding in Brazil is mostly performed intensively with animals reared in confinement. This allows the use of cemented pen with high animal density, however, abnormal behaviours are commonly observed because of the animals being unable to perform their natural behaviours, and their well-being is impaired [1].

In the nursery phase, many simultaneous stress situations occur, such as the separation of the piglets from the mother, a change from a liquid to solid diet, a change of environment, and a mixing of litters [2]. However, by monitoring animal behaviours, we can identify the interaction between their biological needs and their responses to environmental conditions, so we can use the behaviours to assess the adaptability of the animals to the environment.

In this sense, the social and physical environments the animals are exposed to during the nursery phase can alter their behaviour and stress levels [2]. The use of suitable objects for environmental enrichment can reduce stress, for example, the use of aromatised objects reduced the duration of agonistic behaviours of the piglets and the exchange of fragrances increased the interest of the animals in the new object [3], reinforcing the notion that piglets alter their behaviours according to the environmental stimuli they receive.

When compared to the behaviours of trapped or loose animals, it is observed that animals raised in a non-intensive system are more active and perform more natural behaviours, such as eating fodder and grounding, than those that are intensively reared, which tend to increase their frequency of sniffing, nibbling, pushing, and manipulate the tail, compared to animals raised outdoors. In addition, pasture in outdoor environments might play an important role in reducing aggressive behaviours [4].

Thus, the pressure from society and the international community to adapt the productive system to the ideal conditions of animal welfare and the challenges that exist for these changes in the productive system highlight the need for research to evaluate animal behaviour and well-being. Therefore, the objective of this work is to analyse the effect of environmental enrichment on the behaviour of nursery piglets.

## MATERIALS AND METHODS

The present research was approved by the Ethics Commission on the Use of Animals (CEUA), Federal Technological University of Paraná, Dois Vizinhos, PR, Brazil, protocol 2016-034.

The experiment was conducted on a private commercial farm located inside the city of Dois Vizinhos, Paraná, Brazil, at a latitude of 25° 41′ 05″ S, a longitude of 53° 07′ 13″ W, and at an altitude of 546 m. According to the classification of Köppen, the climate in this region is considered humid subtropical (Cfa or Mesothermal), with temperatures between −3°C and 18°C in the cold months and approximately 22°C in the hot months.

The development of the research was carried out in a nursery room with a total area of 178 m^2^ (17.4×10.23 m), a raised canvas lining 3m high, and two side curtains. The handling of these curtains (opening and closing) was done manually, according to the temperature and ventilation. The room was composed of 10 pens, with a polyethylene floor. However, for the experiment, only nine pens with capacity for approximately 50 animals were used, which were divided between 25 males and 25 females. Each pen was equipped with a semiautomatic feeder and nipple drinker.

Approximately 450 female and uncastrated male hybrid piglets were used during postweaning (21 days of age), weighing approximately 6 kg. For the behavioural evaluation, 10 piglets were randomly selected per pen.

The animals were submitted to 7 days of adaptation to the facilities to establish a social hierarchy and so that the management of standardization of the animals in the pen was only carried out during this period, in order to avoid changes in the groups during the evaluations.

All animals received the same nutritional management during the experimental period, with rations of equal nutritional levels provided with each treatment. The rations were supplied *ad libitum* and the types of rations determined according to the age of the piglets. In the first 10 days, the feed supplied was pre-initial 1, pelleted, and then changed to pre-starter ration 2, pelleted, and from the 25th day until the end of the experiment, initial ration, also pelleted, was supplied.

Previous tests had been carried out to determine the type of environmental enrichment, how it would be used, and also to verify the durability of the enriching objects, since the basic requirement was for the object to survive the whole experimental period without needing to be replaced.

The treatments were divided as follows:

- Control treatment: pen without environmental enrichment;
- CEE treatment: continuous environmental enrichment with rubber balls throughout the experimental period;
- EEWB treatment: environmental enrichment with washed balls, with rubber balls removed daily for washing throughout the experimental period.

In the CEE and EEWB treatments, four rubber balls were distributed per pen. The management of the rubber balls in the EEWB treatment consisted of removing them daily, at 8 a.m. for washing with neutral soap and running water and then replacing them in the pen. The purpose of this management was to renew the object, causing an impact of novelty. In the CEE treatment, the balls were not washed for the entire experimental period.

Four days of behavioural evaluation were performed once a week (from 08:00 to 18:00 hours), using images captured with the assistance of video cameras that were installed in the lining and walls of the premises, allowing a greater field of vision. The images were stored on a digital video recorder (DVR) apparatus and analysed after the end of the experiment.

The behavioural observation method was used: animal-focal sampling, with instantaneous recording. To record the instantaneous behaviour, 10 piglets per pen (20% of the pen), considered as focal objects, were randomly selected. These animals were identified in the dorsal region by the numbers from 1 to 10, with permanent pen, for later identification on the videos. For this, a 15-minute sampling interval was used; the images were paused every 15 minutes and the behaviours annotated in spreadsheets.

The behaviours were evaluated based on an ethogram adapted from the methodologies proposed by Camerlink et al [5], Guy et al [6], and O’Driscoll et al [7], according to Table 1.

The number of interactions with the objects in treatments with CEE and with EEWB was stipulated by behaviour sampling, with continuous recording for 10 consecutive hours (from 08:00 to 18:00 hours), considering all the animals present in each pen. For this, the images stored on the DVR apparatus were analysed and for each image, any animal in the pen with a rubber ball was counted as an interaction.

In order to evaluate the behavioural data, the percentages of frequencies were determined for each behaviour of the ethogram. Statistical analyses were performed using the statistical package SAS (SAS Inst. Inc., Cary, NC, USA). The frequency averages were submitted to a non-parametric analysis through PROC NPAR1WAY and compared using the Bonferroni test, where p-values less than 0.05 were considered significant. A Pearson correlation analysis was performed using the PROC CORR procedure.

## RESULTS

**Figure 1:**
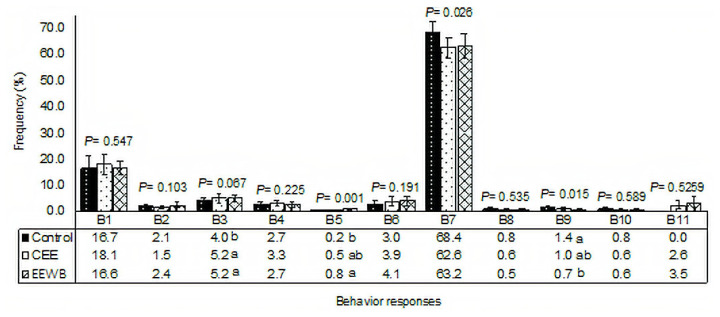
Observed frequency of behavioural responses assessed for each treatment. CEE, continuous environmental enrichment; EEWB, environmental enrichment with washed ball; B1, eating; B2, drinking; B3, getting around; B4, poking with the muzzle around the pen; B5, play; B6, standing or sitting leisure; B7, lying; B8, agonistic interaction; B9, belly nosing; B10, oral manipulation; B11, interacting with the enriching object.

The results obtained in the experiment are presented in Figure 1. A statistical difference (p = 0.001) was observed for the play (B5) behaviour of the animals; pigs of the EEWB treatment group had a higher frequency of this behaviour than the control treatment group without environmental enrichment. The CEE and control treatments did not differ statistically.

Pigs of the control treatment group had a higher frequency of the lying (B7) behaviour (p = 0.026) than those of the EEWB and CEE treatment groups. The EEWB and CEE treatments did not differ from each other. For the variable belly nosing (B9), animals of the control treatment group presented a higher frequency of this behaviour than pigs of the EEWB treatment group (p = 0.015) and did not differ statistically from the CEE treatment group. There was a tendency for an increase in the frequency of behaviour getting around (B3) (p = 0.067) when the animals received the control treatment compared to the other treatments.

A correlation analysis was performed taking into consideration each treatment. For the control treatment, there was a significant negative correlation between the eating (B1) and standing or sitting leisure (B6) behaviours (r = −0.662, p = 0.019), and eating and lying behaviours (r = −0.695, p = 0.012) (Table 2).

For the CEE treatment, there was a negative correlation between the behaviours eating and lying (r = −0.711; p = 0.010), drinking and agonistic interaction (r = −0.705; p = 0.011), standing or sitting leisure and agonistic interaction (r = −0.585; p = 0.046), and poking with the muzzle around the pen and belly nosing (r = −0.650, p = 0.022). There is a positive correlation between the behaviours drinking and belly nosing (r = 0.665, p = 0.018), and poking with the muzzle around the pen and oral manipulation (r = 0.654, p = 0.021) (Table 3).

The EEWB treatment had a negative correlation between eating and lying (r = −0.681, p = 0.015), drinking and poking with the muzzle around the pen (r = −0.629, p = 0.029), drinking and oral manipulation (r = −0.654, p = 0.021 ), getting around and lying (r = −0.718, p = 0.009), ludic and lying (r = −0.775, p = 0.003), and lying and belly nosing (r = −0.622, p = 0.031). There is a positive correlation between eating and ludic (r = 0.699, p = 0.012), eating and belly nosing (r = 0.585, p = 0.046), and getting around and standing or sitting leisure (r = 0.590, p = 0.044). For the other analyses and correlations, there was no effect of the treatments (Table 4).

## DISCUSSION

The presence of environmental enrichment objects and substrates motivates the natural behaviours of swine and positively influences adaptation and the social behaviour between individuals [8].

Therefore, the increase in the frequency behaviour of B5 (recreational) in animals that were receiving treatment EEWB can be attributed to the fact that environmental enrichment stimulated play of the EEWB pigs. This active behaviour stimulates the mesolimbic dopamine centre of the brain causing a sensation of well-being and happiness in the animal [9].

The play behaviour has been touted as a health indicator, since usually during stressful events, such behaviour disappears [10]. Thus, it can be said that there was an improvement in the welfare of the animals in the EEWB treatment group compared to the control treatment and CEE groups, possibly because washing the balls daily aroused a greater interest of the animals.

According to Kornum and Knudsen [11], pigs have a very sensitive sense of smell and recognise the environment through this stimulus. Thus, it is suggested that the smell of the enriching object drove the attraction by the pigs. When the balls were washed and returned to the pen, the renewal of the odour stimulated contact by the animals due to its novelty.

Pigs quickly lose interest in an object of environmental enrichment and the degree of novelty has a direct influence on this behavior [6,12].

The pigs played more with new enriching objects that were added in the pen because they were washed every day. After they became dirty, the animals did not exploit them as much [13].

Studying the interest of piglets in clean and dirty sisal rope, and found that the animals were more interested in the ropes that were suspended above the ground and had no contact with faeces [14].

Quantifying the time in minutes of the interaction of piglets with tyres that were washed daily and replaced in bins and unwashed tyres, observed that the act of washing the object had a positive effect on the contact time by the animals due to the changes related to hygiene and odour [15].

When observing the results obtained for the behaviour B7 (lying down), it is suggested that the presence of environmental enrichment made the animals more active, devoting more time to interacting with the balls than to lying down.

This result corroborates that of Machado et al [1], since they observed that piglets in the termination phase kept in a sterile environment remained lying down 67.72% of the time, whereas the animals that received objects of CEE on alternate days and continuously during the day spent 58.31%, 59.27%, and 65.31% of their time lying down, respectively.

In a study by Vanheukelom et al [16], piglets that had access to peat as environmental enrichment slept less than those did housed without peat.

Of all production animals, pigs spend most of their time sleeping or resting [17]. Piglets weaned at 21 days submitted to treatment without environmental enrichment laid down 69.22% of the time however, this value did not differ statistically from treatments with an enriched environment [13].

With regard to the reduction in B9 behaviour in the EEWB treatment group, the piglets were likely to redirect their attention to the rubber balls and spend more time playing with them, thus lessening their interest in manipulating another animal.

In this study also observed statistically significant differences for this characteristic between piglets submitted to environmental enrichment and those maintained in a sterile environment [18].

Environments enriched with peat and straw allowed the animals to spend more time exploring the substrates, and consequently they performed fewer harmful behaviours, such as belly nosing [19].

According to Guy et al [6], sterile environments favour the development of abnormal behaviours. Thus, one way to reduce these problems in the nursery phase is to encourage piglets to perform their primary natural behaviour, which is exploration of the environment. According to Bench and Gonyou [18], any environmental enrichment can promote this behaviour and is an efficient way of controlling behavioural defects.

When we evaluated the correlations within each treatment, we observed that in the control treatment, there was a negative correlation between the behaviours B1 and B6, and B1 and B7, which indicated that the animals were either idle, lying down, or eating. This is not surprising, since these are antagonistic activities. Moreover, piglets kept in a sterile environment remained lying down 67.72% of the time, and this value was only reduced by adding enriching objects to the environment [1].

For the CEE treatment, a negative correlation between B1 and B7 was again observed, as well as for B8 and B2, B6, and B9, i.e. the agonistic behaviour (B8) can be reduced when using an enriching object that increases the performance of behaviours B2, B6, and B9. For this treatment, positive correlations were observed between behaviours B2 and B9, B3 and B8, and B4 and B1. When observed together, it is notable that the most active animals, due to the introduction of the object, end up performing these activities. This is important when trying to understand the behaviour of piglets at this stage, since the tendency of animals to explore environments is natural, and when there is no stimulating object, these activities are instead directed towards other partners [3].

The EEWB treatment showed a negative correlation between the behaviours B1 and B7, B2 and B4, B2 and B10, B3 and B7, and B7 and B9, and a positive correlation between B1 and B5, B1 and B9, and B3 and B6. These correlations indicate that animals that spend more time eating, drinking, walking in the pen, and lying down, are less likely to perform agonistic behaviours, such as belly nosing and oral manipulation, and this occurs in the presence of an enriching object supplied intermittently.

It was concluded that the use of an enriching object and washing it daily led to an improvement in animal welfare and altered the correlation between the evaluated behaviours compared to the animals that did not have environmental enrichment.

## Figures and Tables

**Table 1:** Ethogram of work to evaluate the behaviours of nursery piglets with environmental enrichment

Behaviour	Description
Eating (B1)	Animal at the feeder presenting feed intake
Drinking (B2)	Animal at the drinking fountain showing water intake
Getting around (B3)	Animal in walking motion by the pen
Poking with the muzzle around the pen (B4)	Animal touching, sniffing or poking around the enriching object for at least 5 second
Play (B5)	Animals playing with each other, running, or jumping around the pen
Standing or sitting leisure (B6)	Animal standing or sitting, without any activity
Lying (B7)	Animal lying with the body in contact with the floor, with the eyes open or closed
Agonistic interaction (B8)	Interacting aggressively with another animal (pushing or biting)
Belly nosing (B9)	Animal pressing the belly of another animal with the muzzle, repetitively similar to breastfeeding
Oral manipulation (B10)	Animal biting the tail or ear of another animal
Interacting with the enriching object (B11)	Animal touching, smelling, or nibbling the enriching object for at least 5 seconds

**Table 2:** Correlation coefficients obtained between the behavioural responses evaluated for the control treatment

Behaviour	B1	B2	B3	B4	B5	B6	B7	B8	B9
B2	0.0261)	-	-	-	-	-	-	-	-
	0.9372)								
B3	−0.385	0.257	-	-	-	-	-	-	-
	0.217	0.421							
B4	0.088	0.159	−0.074	-	-	-	-	-	-
	0.785	0.622	0.819						
B5	−0.209	0.000	−0.257	−0.026	-	-	-	-	-
	0.514	1.000	0.420	0.935					
B6	−0.662	0.483	0.371	0.318	0.445	-	-	-	-
	0.019	0.112	0.235	0.313	0.147				
B7	−0.695	−0.498	−0.124	−0.392	0.158	0.044	-	-	-
	0.012	0.099	0.701	0.208	0.625	0.892			
B8	−0.347	−0.013	0.414	−0.149	0.263	0.464	−0.087	-	-
	0.268	0.968	0.181	0.644	0.409	0.129	0.789		
B9	−0.451	0.043	0.425	0.290	−0.445	0.191	0.066	0.113	-
	0.141	0.895	0.169	0.361	0.147	0.552	0.838	0.726	
B10	0.126	−0.032	−0.176	−0.423	0.229	−0.038	−0.182	0.548	−0.091
	0.696	0.922	0.583	0.171	0.474	0.908	0.571	0.065	0.780

B1, eating; B2, drinking; B3, getting around; B4, poking with the muzzle around the pen; B5, play; B6, standing or sitting leisure; B7, lying; B8, agonistic interaction; B9, belly nosing; B10, oral manipulation

1) Correlation coefficient.

2) p-value.

**Table 3:** Correlation coefficients obtained between the behavioural responses evaluated for the continuous environmental enrichment treatment

Behaviour	B1	B2	B3	B4	B5	B6	B7	B8	B9	B10
B2	0.0201)	-	-	-	-	-	-	-	-	-
	0.9502)									
B3	0.197	−0.370	-	-	-	-	-	-	-	-
	0.539	0.236								
B4	0.153	0.193	0.256	-	-	-	-	-	-	-
	0.635	0.548	0.421							
B5	−0.332	0.060	0.378	−0.096	-	-	-	-	-	-
	0.292	0.854	0.226	0.767						
B6	−0.392	−0.023	−0.360	−0.549	−0.032	-	-	-	-	-
	0.208	0.944	0.250	0.065	0.921					
B7	−0.711	−0.190	−0.392	−0.524	0.113	0.202	-	-	-	-
	0.010	0.553	0.208	0.080	0.727	0.530				
B8	0.167	−0.705	0.640	0.192	0.106	−0.585	0.026	-	-	-
	0.604	0.011	0.025	0.550	0.743	0.046	0.935			
B9	−0.415	0.665	−0.326	0.145	0.029	0.068	0.126	−0.650	-	-
	0.180	0.018	0.300	0.652	0.929	0.833	0.697	0.022		
B10	0.264	0.054	0.083	0.654	−0.145	−0.181	−0.512	0.048	0.105	-
	0.407	0.868	0.799	0.021	0.652	0.573	0.089	0.883	0.746	
B11	−0.502	0.290	−0.404	0.228	0.027	0.286	0.013	−0.454	0.433	−0.048
	0.097	0.361	0.192	0.476	0.933	0.367	0.968	0.139	0.160	0.883

B1, eating; B2, drinking; B3, getting around; B4, poking with the muzzle around the pen; B5, play; B6, standing or sitting leisure; B7, lying; B8, agonistic interaction; B9, belly nosing; B10, oral manipulation; B11, interacting with the enriching object.

1) Correlation coefficient.

2) p-value.

**Table 4:** Correlation coefficients obtained between the behavioural responses evaluated for the environmental enrichment with washed balls treatment

Behaviour	B1	B2	B3	B4	B5	B6	B7	B8	B9	B10
B2	−0.2341)	-	-	-	-	-	-	-	-	-
	0.4642)									
B3	0.322	0.258	-	-	-	-	-	-	-	-
	0.308	0.417								
B4	0.380	−0.629	0.075	-	-	-	-	-	-	-
	0.223	0.029	0.817							
B5	0.699	−0.196	0.327	0.512	-	-	-	-	-	-
	0.012	0.541	0.300	0.089						
B6	−0.277	0.371	0.590	−0.073	0.083	-	-	-	-	-
	0.383	0.236	0.044	0.821	0.798					
B7	−0.681	0.072	−0.718	−0.454	−0.775	−0.334	-	-	-	-
	0.015	0.824	0.009	0.138	0.003	0.288				
B8	−0.178	−0.384	−0.378	0.470	−0.208	−0.356	0.353	-	-	-
	0.581	0.218	0.226	0.123	0.516	0.255	0.260			
B9	0.585	−0.308	0.348	0.199	0.552	0.083	−0.622	−0.247	-	-
	0.046	0.331	0.268	0.535	0.063	0.797	0.031	0.439		
B10	0.178	−0.654	0.080	0.556	0.084	−0.441	−0.124	0.571	0.175	-
	0.580	0.021	0.804	0.061	0.794	0.151	0.702	0.053	0.586	
B11	−0.247	−0.076	−0.190	−0.182	−0.002	0.016	−0.107	−0.419	0.037	−0.134
	0.440	0.814	0.554	0.571	0.994	0.961	0.741	0.175	0.910	0.678

B1, eating; B2, drinking; B3, getting around; B4, poking with the muzzle around the pen; B5, play; B6, standing or sitting leisure; B7, lying; B8, agonistic interaction; B9, belly nosing; B10, oral manipulation; B11, interacting with the enriching object.

1) Correlation coefficient.

2) p-value.
